# Not Just for Dancing? A Content Analysis of Concussion and Head Injury Videos on TikTok

**DOI:** 10.3389/fspor.2021.692613

**Published:** 2021-10-27

**Authors:** Peyton N. Carter, Eric E. Hall, Caroline J. Ketcham, Osman H. Ahmed

**Affiliations:** ^1^Department of Exercise Science, Elon University, Elon, NC, United States; ^2^Elon BrainCARE Research Institute, Elon University, Elon, NC, United States; ^3^Department of Physiotherapy, University Hospitals Dorset NHS Foundation Trust, Poole, United Kingdom; ^4^School of Sport, Health and Exercise Science, University of Portsmouth, Portsmouth, United Kingdom

**Keywords:** social media, concussion, head injury, healthcare education, public health

## Abstract

Social media platforms are an accessible and increasingly used way for the public to gather healthcare-related information, including on sports injuries. “TikTok” is currently one of the fastest-growing social media platforms worldwide, and it is especially popular amongst adolescents and young adults. The widespread use and popularity of TikTok suggests that this platform has potential to be a source for healthcare information for younger individuals. The aim of this study was to gain a preliminary understanding of the concussion/head injury-related information on TikTok, and to gauge if TikTok could serve as a platform for concussion education. This exploratory study used a systematic search strategy to understand more about how concussion is being portrayed through TikTok videos. Using the keywords “concussion” and “head injury,” 200 videos were downloaded from TikTok and 43 videos were excluded. Of the 92 videos retrieved using the keyword “concussion,” 95% (*n* = 88) had more than 100,000 views and 6% (*n* = 10) had been viewed more than 10 million times. Over half, 54% (*n* = 50) of the “concussion” videos depicted individuals “playing around” and getting hit in the head, whilst only 1% (*n* = 1) of the TikTok videos were categorized as “explaining concussion facts.” The large numbers of views of concussion-related TikTok videos demonstrates the popularity of this platform and indicates that healthcare organizations should consider TikTok as a potential means for concussion education amongst younger individuals.

## Introduction

The intersection of the social media and sports medicine worlds is still in its relative infancy, although there are increasing examples of how these fields are associated. This is unsurprising given the ubiquity of social media usage globally; whereas in 2005 there were 5% of U.S. adults using at least one social media site (Pew Research Center, 2019a), by 2019 this had increased to 72% (Pew Research Center, 2019a). There are now 3.5 billion social media users worldwide, with this number projected to increase to 4.41 billion users by 2025 (Statista, 2020), and it is suggested users can spend an average of 3 h per day on social media platforms and messaging apps (Oberlo, 2020).

The “coming together” of social media and sports medicine is happening on several levels. Clinicians are using social media to provide themselves with an online presence (Shurlock et al., 2018), and students are using it as an education platform (Rigamonti et al., 2020). Elite athletes have also been shown to obtain some positive support from social media messages during major sporting events (Hayes et al., 2020), and to use social media platforms during their injury recovery period for social support (Nankervis et al., 2018).

One of the “hot topics” in sports medicine at present is sports-related concussion. Concussion, most recently defined as a traumatic brain injury induced by biomechanical forces (McCrory et al., 2017), has been the subject of extensive discourse in the academic literature (Alla et al., 2011) and the mainstream press (Ahmed and Hall, 2017). Appropriate knowledge regarding concussion is imperative to its successful management (McCrea et al., 2017), however many key stakeholder groups (including athletes, coaches and trainers) have been shown to either have a lack of concussion knowledge (Sullivan et al., 2012a), incorrect concussion beliefs (White et al., 2014), or under-report concussions (Conway et al., 2020). To combat this, there have been numerous campaigns across different sports to highlight awareness and understanding of the correct management of concussion (Centers for Disease Control and Prevention, 2020).

Social media has also been used as a vehicle to promote concussion information. Ahmed et al. (2010) assessed how concussion-related information was shared on Facebook, whilst a similar methodology was used to examine concussion-related tweets by Sullivan et al. (2012a). Findings from these two studies suggested that Facebook was primarily used for peer support (termed “iSupport”), whilst concussion-related tweets were most commonly categorized as “news” (33%), “sharing personal information/situation” (27%), and “inferred management” (13%). Images of concussion shared on the social media platforms Pinterest, Instagram and Flickr have also been evaluated (Ahmed et al., 2016), with the majority of images sharing a concussion-related incident (33%).

Concussion-related videos on social media were the subject of the study by Williams et al. (2014), where these authors systematically retrieved 434 videos and included the 100 most-viewed videos for analysis. There were a variety of themes represented by the videos included in this study (e.g., 37% portrayed a recreational/sporting injury, 24% of the videos were news items), however only 11% of videos were categorized as “educational” and only 4 videos recognized by an academic source. Whilst YouTube may be a viable platform to share concussion-related information, the content, quality, and source of these videos is variable.

Another mainstream social media video platform is TikTok, currently one of the fastest-growing social media platforms worldwide (Forbes, 2020). Primarily used for entertainment purposes, its major demographic user group are 16–24 years old (which compromise 41% of all users) (Beer, 2019). TikTok's appeal lies in the “snappy” nature of its content, with users permitted to post videos of between 1 and 60 s length (Slate, 2018). The app now has an extensive global reach, a reported 1 billion monthly users and over 2 billion downloads worldwide (Wallaroo, 2021).

Given the recent emergence of TikTok as a global platform, examples of its use for healthcare-related purposes are limited. Much of the literature focused on TikTok and healthcare is related to the current COVID-19 pandemic. Chen et al. (2021) assessed citizen engagement with the National Health Commission of China during the COVID-19 pandemic, finding that shorter videos with longer video titles had the greatest engagement. The study of Basch et al. (2021) highlighted the use of TikTok in promoting face mask use to prevent the spread of the virus, reporting that videos which featured humor and dance were factors in driving engagement. The popularity and functionality of TikTok was also noted by Comp et al. (2020), who reinforced that TikTok has benefits for both public health and medical education.

The importance of knowledge transfer in education for sports concussion has been well-documented (Provvidenza and Johnston, 2009) as has the role that social media can play in this process (Provvidenza et al., 2013), particularly with adolescents (Kollia et al., 2018). Given the global popularity of TikTok and its engaging video-based format, it has the potential to be a valuable source of concussion information and education. To date however, there has been no exploration of concussion-related content on TikTok. The aim of this study therefore was to gain a preliminary understanding of the concussion/head injury-related information on TikTok, and to gauge if TikTok could serve as a platform for concussion education.

## Methods

### TikTok

The TikTok platform was used to conduct a systematic review of the videos posted on this social media site in relation to concussions. Similar to previous studies evaluating concussion information in a variety of social and mainstream media platforms, a snapshot approach was used (Sullivan et al., 2012b; Williams et al., 2014; Ahmed and Hall, 2017). Table 1 provides an outline of some of the key terms related to the functionality present in TikTok.

**Table 1:** TikTok terminology.

**Terminology**	**Description**
Verified^a^ [person (PV) or organization (OV)]	TikTok provides badges to confirm the identities of accounts to help users find legitimate accounts and see authentic content. TikTok believes that this helps with clarity and build community.
Hashtags	Hashtags (#) are used on TikTok (and other social platforms) to group videos and could include helping users share videos, to find videos and to help grow their audience.
Trending Hashtags^b^	Specific hashtags that are popular at the moment and may help bring attention to the posts of the user (e.g., “#fyp, #foryoupage” etc.). These trending hashtags are constantly in flux.

a *https://newsroom.tiktok.com/en-us/how-to-tell-if-an-account-is-verified-on-tiktok*.

b *https://influencermarketinghub.com/hashtag-analytics-101-metrics-hashtags-and-analytics-tools/*.

### Pilot Testing

The researchers conducted initial pilot testing (22 June 2020) by examining a total of 15 videos using the terms: “concussion symptoms,” “concussion facts,” “concussion education,” and “concussion.” The links from these videos were put into a spreadsheet and some initial coding of these videos took place to help refine coding of the final videos (see “Data analysis”).

### Search Strategy for Study

A systematic strategy was used to collect the videos at the time of search (12 pm CST on 17 July 2020). The TikTok application was used to select the links for the videos. The first 100 videos were used for the search terms “concussion” and “head injury” for future analysis. All the links were placed into a spreadsheet for future analysis.

### Inclusions and Exclusion Criteria

The inclusion criteria for this study were that videos must be in English and involve in some way a head injury. The exclusion criteria included: videos not in English and not involving head injury. Duplicate videos and videos that could not be accessed when coding occurred (e.g., deleted or private account) were also not included in the analysis.

### Data Analysis

From the TikTok platform, some general descriptors of the video (e.g., number of likes and number of views) are reported. This data was captured to help get a sense of reach of the videos and who was posting videos. At the time of the initial downloading, the number of likes and views was recorded. On the day that data was coded, the number of forwards, number of comments, hashtags, the year the video was uploaded, and the source of content were recorded. These descriptors are presented in Table 2. The descriptors of likes, views and comments were separated, tallied, and reported as a percentage of total. The descriptors of hashtags, year uploaded, and sources of content were tallied and reported as a percentage of total.

**Table 2:** TikTok video descriptors for head injury and concussion labels (videos tallied for each category and reported as a percentage of total/number of verified people (PV) or verified organization (OV) reported).

	**Head injury (*n* = 65)**	**Concussion (*n* = 92)**
Number of views		
> 10 million	0 (0%)	10 (11%) (3PV)
1–10 million	4 (6%) (1PV; 1OV)	44 (68%) (1OV; 5PV)
100k−1 million	7 (11%) (1PV)	34 (37%) (1OV; 3PV)
10–100k	19 (29%)	4 (4%)
<10k	35 (54%)	0 (0%)
Number of likes		
>1 million	0 (0%)	12 (13%)
100k−1 million	6 (9%)	51 (55%)
10–100k	8 (12%)	26 (28%)
1–10k	16 (25%)	3 (3%)
<1k	35 (54%)	0 (0%)
Number of forwards		
>100k	0 (0%)	5 (5%)
50–100k	0 (0%)	8 (9%)
10–50k	0 (0%)	15 (11%)
1–10k	5 (8%)	44 (48%)
<1k	60 (92%)	20 (22%)
Number of comments		
>10k	0 (0%)	11 (12%)
1–10k	4 (6%)	38 (41%)
<1k	55 (85%)	42 (46%)
None	6 (9%)	1 (1%)
Hashtags		
Concussion	0 (0%)	5 (5%)
Head injury	12 (19%)	0 (0%)
Trending	38 (59%)	67 (73%)
None	15 (23%)	20 (22%)
Sources		
Person	61 (94%)	79 (86%)
Verified person	2 (3%)	11 (12%)
Organization	1 (2%)	0 (0%)
Verified organization	1 (2%)	2 (2%)
Year uploaded		
2018	2 (3%)	0 (0%)
2019	19 (29%)	44 (11%)
2020	44 (68%)	48 (52%)

The lead author (PNC) viewed the videos multiple times to accurately classify content of the videos. Videos were originally classified on broad content areas: Recreational/Sporting Injury, News, Educational, Commercial, Personal and Other. These categories were originally chosen based on previous research by Williams and colleagues assessing YouTube videos (Williams et al., 2014). Videos were then classified into more specific content topics. These topics were determined by all authors and included: video footage of sport injury; playing around/hit in the head; personal story about injury; joking/acting/faking injury; diagnosed concussion/head injury; downplay/bragging about symptoms; and concussion facts. The classification of all videos was confirmed with an outside reviewer. Where discrepancies occurred in classification by the outside reviewer, two authors (CJK and EEH) worked through these discrepancies. The classification of content themes and topics are presented in Table 3. Each video was only included in one classification category, and then tallied and reported as a percentage of the total.

**Table 3:** TikTok video content themes and topics (tallied and reported as percentage of total).

	**Head injury** ** (*n* = 65)**	**Concussion** ** (*n* = 92)**
Broad content themes		
Rec Sport/Inj	5 (8%)	25 (27%)
News	0 (0%)	0 (0%)
Education	2 (3%)	1 (1%)
Commercial	0 (0%)	0 (0%)
Personal	45 (69%)	41 (45%)
Other	13 (20%)	25 (27%)
Content topics		
Video footage of sport injury	1 (2%)	15 (16%)
Playing around, hit head	17 (26%)	50 (54%)
Personal story	25 (39%)	8 (9%)
Joking, acting, faking	13 (20%)	12 (13%)
Diagnosed concussion/head injury	7 (11%)	4 (4%)
Downplay/brag about symptoms	0 (0%)	2 (2%)
Concussion facts	2 (3%)	1 (1%)

## Results

**Figure 1:**
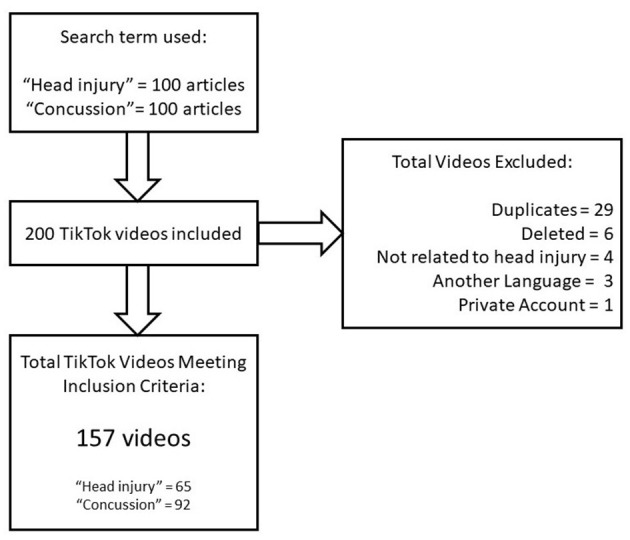
Flowchart showing how videos were selected.

After the initial downloads of 100 videos for Head Injury and 100 videos for Concussion, 157 total videos met inclusion criteria (head injury = 65; concussion = 92; see Figure 1). These videos were analyzed for descriptor information (Table 2) and content (Table 3). Concussion videos overall had more engagement measured by views, likes, forwards, comments, and hashtags. Ten concussion videos had over 10 million and 44 more over 1 million views suggesting impact/influence is broad for the concussion label. The number of videos increased from 2018 to 2020, following the trend of the platform.

While engagement and reach of the videos showed some breadth, the question of the content and source of the videos was of interest to provide insight on the platform as an educational possibility (Table 2). Most videos were personal with only 3 total videos being educational in content. Similarly, most videos were uploaded by individual people, many who were not verified users. Only 4/157 videos were uploaded by an organization, 3 by a verified organization. Finally, the topics of videos were analyzed with content mostly around playing around and hitting their head, discussing a personal story or situation, or joking, acting or faking a head injury or concussion. Only 11/157 videos referenced a diagnosed head injury (*n* = 7) or concussion (*n* = 4) and only 3/157 videos covered concussion facts (head injury = 2; concussion = 1). One of the educational/concussion fact videos fell under both the “head injury” and “concussion” hashtag and was uploaded by a verified organization. This video had 1.2 million views, 900 comments, and 2,065 forwards. It is the only video that came up in the top of our search for both head injury and concussion labels and included educational and factual concussion information.

## Discussion

Our study has reinforced that as well as being a popular social media platform for entertainment, TikTok is also being used to share information on concussion and head injury. The majority of videos retrieved in this study showed a light-hearted approach to the topic of concussion, and only 2 of the videos included in our study were categorized as “educational” videos with 1 being viewed 1.2 million times with both the head injury and concussion labels and the other one only had 253 views. Given the reach of the one highly-viewed educational video, there is potential for TikTok to be harnessed in order to share concussion education content with its young audience.

There were a significant amount of videos with hashtags in the caption, however very few videos had hashtags relating to concussion or head injury. The majority of videos (*n* = 105) had “trending” hashtags (defined in Table 1) in their caption, which may have been responsible for the number of views that these videos had. Utilizing hashtags/trending hashtags in a nuanced manner would be a constructive means of boosting the reach of educational videos on TikTok, and help to propagate best-practice concussion information to the widest possible audience. In order to achieve this, collaborative approaches using the knowledge and expertise of the younger generations that are familiar with the use of TikTok (e.g., “Generation Z”/“Generation Alpha”) is warranted.

At the time of writing, there were no comparable studies relating to the use of TikTok in the domain of sports medicine. The work of Zheng et al. (2021) provided an example of using a quality control measure (the DISCERN tool) to provide an assessment of TikTok videos related to acne. The low overall content quality of videos in this study suggested that dermatologists should consider “plugging the information gap” for this condition on TikTok. Although there was no formal evaluation of the quality of concussion/head injury-related videos in our study, many of the videos retrieved appeared to be of a low content quality (i.e., did not promote best-practice recommendations related to recovery following concussion). In keeping with the findings of Lovett et al. (2021) who recommended radiologists adopt TikTok to create clinical content for professional engagement, we propose that sports medicine clinicians should consider the use of this platform to share best-practice concussion information in a similar manner.

Out of the 157 videos found, 16 were videos from a verified account. These videos fell into the top view categories, likely due to the high number of followers of these accounts. This suggests that TikTok has the potential to be a platform to show concussion and head injury education, especially if using brands (e.g., NCAA, NFL, NBA) or “influencers” with verified accounts such as pro-athletes. These accounts would be a key aspect in terms of the information being spread throughout TikTok users. Additionally, concussion education could be ‘sponsored’ by an organization on TikTok. This would provide an incentive for influencers to include concussion education information in their videos, which would reach a large audience of followers (Choudhary et al., 2020).

Given that research on platforms such as TikTok is in its infancy, we acknowledge several limitations to our study. In the absence of any existing framework to capture data from TikTok, we generated our own coding scheme. Although this was not a validated tool, it was based on similar coding schemes from previous concussion-related research on social media platforms (e.g., Ahmed et al., 2010; Sullivan et al., 2012b; Williams et al., 2014) and pilot tested prior to the main data collection being undertaken. The data collection was also limited to the “snapshot” window used, and as such the generalizations may be limited to that period. Given that this time (July 2020) was in the early months of the COVID-19 pandemic, professional/recreational sport was still very restricted globally and this may also have affected the nature of the information collected.

The ubiquity of smartphone use and online access to healthcare information (which has been heightened during the COVID-19 era) has meant that innovative new means have been adopted for health promotion messaging. Our study has indicated that TikTok is one potential platform that could be used to spread accurate concussion information, targeted toward the younger demographic group who are the main users of this platform. Future research could focus on a greater understanding of how Generation Z, the generation who have grown up with access to the internet and are “digital natives” (Pew Research Center, 2019b), view TikTok, and how sports medicine-related educational information could be best disseminated via this platform.

## Conclusion

Our study demonstrates that as well as being used for entertainment, TikTok is also being used to share content on concussion and head injury. There is potential to use this platform to promote concussion awareness and educational material that is accurate, informative, and entertaining. Engaging TikTok to provide evidence-based resources for concussion should be considered, in order to increase concussion awareness and knowledge in a timely and engaging manner.

## Data Availability Statement

The original contributions presented in the study are included in the article/supplementary material, further inquiries can be directed to the corresponding author/s.

## Author Contributions

OHA, CJK, and EEH conceived the idea for this study. PNC undertook the initial data analysis and all authors were involved in final data analysis. All authors contributed toward the writing of this manuscript.

## Funding

PNC was awarded a grant from Elon University Undergraduate Research Program to partially cover open access publication fees.

## Conflict of Interest

The authors declare that the research was conducted in the absence of any commercial or financial relationships that could be construed as a potential conflict of interest.

## Publisher's Note

All claims expressed in this article are solely those of the authors and do not necessarily represent those of their affiliated organizations, or those of the publisher, the editors and the reviewers. Any product that may be evaluated in this article, or claim that may be made by its manufacturer, is not guaranteed or endorsed by the publisher.
